# *Rickettsia felis* in the United Kingdom

**DOI:** 10.3201/eid0908.030314

**Published:** 2003-08

**Authors:** Martin J. Kenny, Richard J. Birtles, Michael J. Day, Susan E. Shaw

**Affiliations:** * University of Bristol, Langford, Somerset, United Kingdom; † University of Liverpool, Leahurst, Neston, Cheshire, United Kingdom

**Keywords:** *Rickettsia felis*, United Kingdom, PCR, flea, cat, dog, zoonosis

**To the Editor:** *Rickettsia felis* is a bacterium transmitted by the cat flea (*Ctenocephalides* *felis*), which also acts as a reservoir by means of transovarial transmission (*1*–*3*). The distribution of *R. felis* is potentially as wide as that of its insect host, and to date, its presence has been confirmed in cat flea populations in North and South America and southern Europe (*4*,*5*). *R. felis* was first identified as a human pathogen in 1994 (*6*), and cases of “flea-borne spotted fever,” which have signs and symptoms of febrile exanthema, have now been reported in the United States, Mexico, Brazil, France, and Germany (*7*,*8*). To our knowledge, reports on the presence of *R. felis*, or indeed any other spotted fever group rickettsia, in the United Kingdom have not been published.

To determine whether *R. felis* is present in the United Kingdom, we surveyed cat fleas collected from dogs and cats seen at veterinary practices in southern England and Northern Ireland. A total of 31 dogs and 79 cats from veterinary practices in Bristol, Dorset, London, Devon, Gloucestershire, Hampshire, and Antrim were included in our study. Fleas were collected by combing these animals for 10 minutes. All fleas from each animal were pooled in 70% ethanol. A total of 316 *Ct. felis* (Bouché, 1835), identified by using accepted morphologic criteria, were obtained, with each animal yielding one to five fleas. DNA was extracted from each of the 110 flea pools by using a standard silica cartridge method (QiaAmp DNA mini kit, QIAGEN Ltd., Crawley, West Sussex, U.K.) using the manufacturer’s instructions for tissue DNA extraction. The presence of rickettsial DNA was determined by using the polymerase chain reaction (PCR) with oligonucleotide primers that target rickettesial *ompB* (*5*) or *gltA* (*2*) genes. Positive control material was cultured *R. felis*. Rigorous controls to limit contamination were carried out, including the use of separate, dedicated rooms for DNA extraction, PCR setup, and gel analysis. Amplification products obtained from *ompB* and *gltA* PCRs were analyzed by using DNA sequencing. Sequences obtained were edited by using BioEdit (available from: URL: http://www.mbio.ncsu.edu/BioEdit/bioedit.html). Similarity to published sequences was determined with the BLAST program (available from: URL: http://www.ncbi.nlm.nih.gov) hosted by the National Centre for Biotechnology Information.

Eighteen flea DNA pools were positive for spotted fever group rickettsia. All 18 yielded PCR products with both *ompB* and *gltA*-targeting PCRs. The *ompB* and *gltA* DNA sequences of all PCR products were 100% identical to those published for *R. felis*, thereby providing evidence for the presence of *R. felis* in fleas collected from >16% of the animals surveyed. PCR-positive fleas were collected from 4 dogs and 14 cats from Bristol, Hampshire, Dorset, and Northern Ireland. Taking into account the number of fleas in each pool, we estimate that 6% to 12% of the fleas collected were infected with *R. felis*.

This study represents the first description of a spotted fever group rickettsia endemic to the United Kingdom. The species detected, *R. felis,* has clear public health implications. The bacterium appears to be widely distributed within the country, infecting a geographically dispersed population of *Ct. felis*. Up to 12% of *Ct. felis* may be infected with *R. felis,* a flea that is by far the most common species of ectoparasite encountered on cats and dogs in the U.K. mainland. Furthermore, *Ct. felis* often feeds on humans.

Clinicians encountering patients with fever or rash (or both) and a history of cat contact or flea bites should consider a diagnosis of *R. felis*. Laboratory confirmation of infection is not easy, but in vitro culture of *R. felis,* and hence material for a serologic assay for the diagnosis of human *R. felis* infections, has recently been described, and serology appears to be an accurate indicator of exposure (*9*). As with other spotted fever group rickettsial infections, molecular diagnostics may provide a useful alternative approach to detecting and identifying *R. felis* in infected tissues. In culture, *R. felis* has been shown to be resistant to erythromycin (unlike other rickettsia), gentamicin, amoxicillin, and trimethoprim-sulfamethoxazole. Thus, infection with this bacterium should be considered in cases of antibiotic-insensitive fever with a rash, especially in young, old, and immunosuppressed persons. The organism is sensitive to doxycyclin, rifampicin, thiamphenicol, and fluoroquinolones (*10*)
